# Supplementary material for: BET inhibitors drive Natural Killer activation in non-small cell lung cancer via BRD4 and SMAD3
Source: Nat Commun. 2024 Mar 22;15:2567. doi: 10.1038/s41467-024-46778-8 (PMC10960013; doi:10.1038/s41467-024-46778-8)
Supplement: Supplementary file 1 — Supplementary Information [file 41467_2024_46778_MOESM1_ESM.pdf]

Supplementary Figure 1

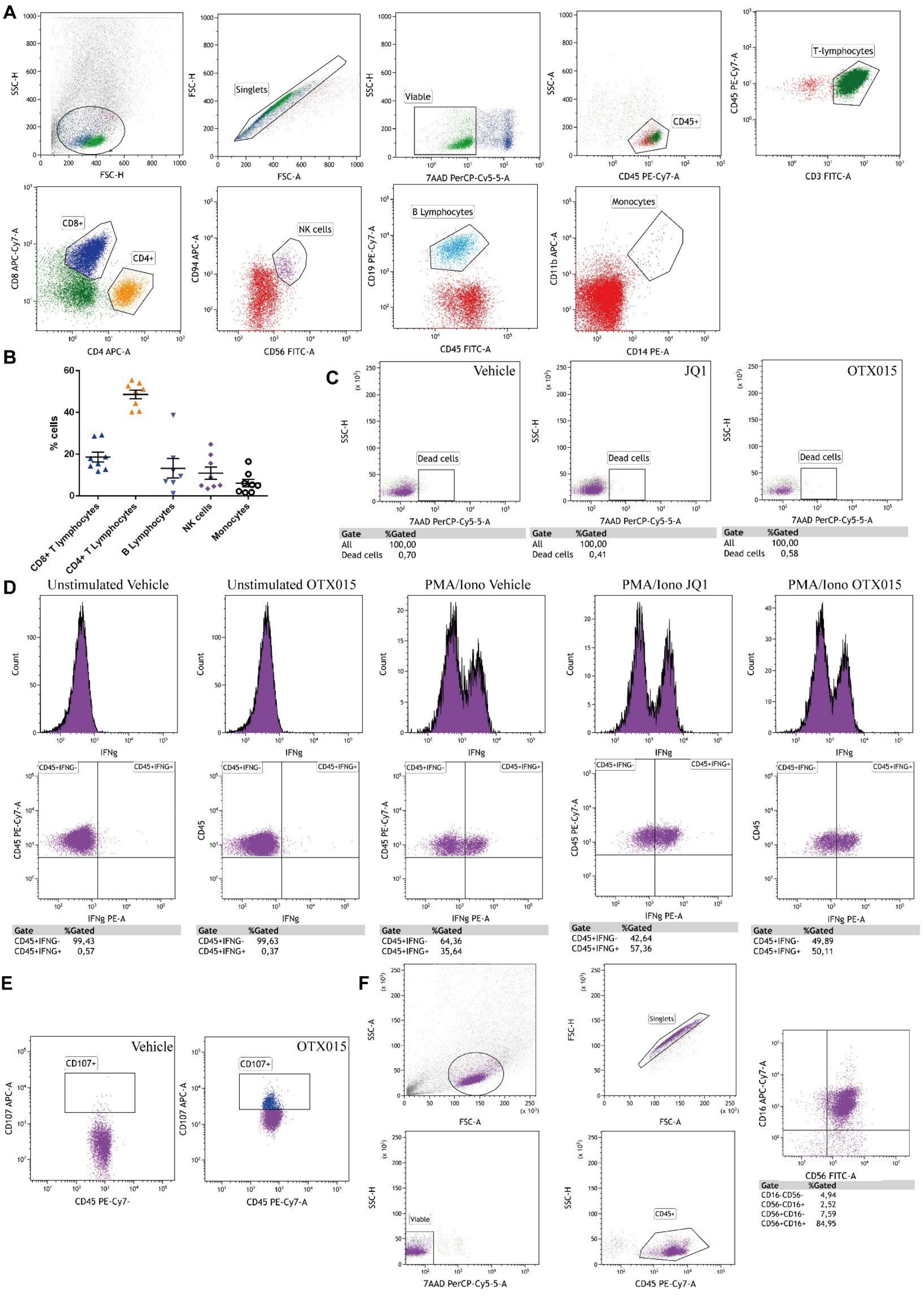

Supplementary Figure 1. A) Gating strategy and flow cytometry analysis of patient-derived immune cells purified from NSCLC surgical specimens. Forward versus side scatter (FSC vs SSC) gating was used to identify cells based on size and granularity. After the exclusion of doublets and dead cells ($7AAD^+$), immune cells were selected for $CD45^+$ expression. The main markers were used to gate T-lymphocytes ($CD3^+$), B-lymphocytes ($CD19^+$), NK cells ($CD56^+$), and Monocytes ($CD14^+$). T-lymphocytes were subsequently classified as T-helper ($CD4^+$) or cytotoxic ($CD8^+$) T cells; **B)** Quantitation of relative amount of each immune cell type in different AD specimens (n=8 patients); **C)** Evaluation of immune cell viability by 7AAD staining in BETi-treated compared to vehicle-treated cells confirmed that the drugs did not affect the number of viable cells in our setting; **D)** Mean fluorescence intensity (MFI) histograms and dot plots of IFN-γ expression in total $CD45^+$ cells (TILs). After PMA/Ionomycin stimulation, BETi increased the number of $CD45^+$IFN-γ^+ cells compared to vehicle; **E)** Increased expression of CD107A in total $CD45^+$ TILs after BETi treatment in co-cultures with NSCLC; **F)** Gating strategy and flow cytometry analysis of purified NK cells from NSCLC surgical specimens. Source data are provided as a Source Data file.

Supplementary Figure 2

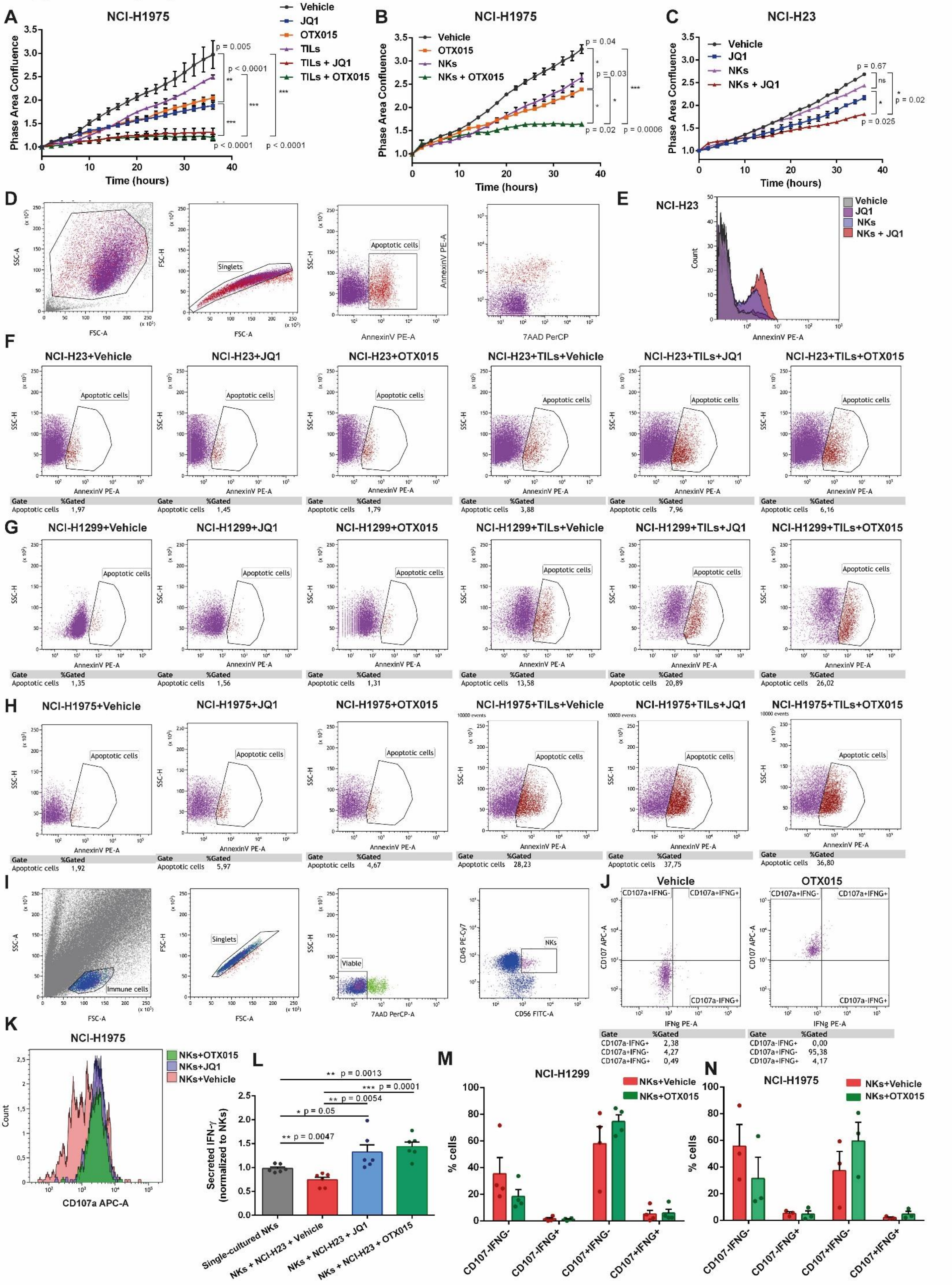

Supplementary Figure 2. A-B) Proliferation assays on NCI-H1975 in co-culture with patient-derived total immune $CD45^+$ cells (TILs) (**A**) or purified NK cells (**B**) and in presence of BETi (1µM JQ1 or OTX015) or vehicle. Tumor cell confluence area was measured by EssenBio Incucyte S3 cell-live imaging (n=3 independent experiments, data are shown as mean ± SEM); **C)** Proliferation assays on NCI-H23 in co-culture with patient-derived NKs in presence of 1µM JQ1 (n=3 independent experiments, data are shown as mean ± SEM); **D)** Gating strategy and flow cytometry analysis of apoptotic tumor cells through AnnexinV staining; **E)** AnnexinV staining of NCI-H23 tumor cells in presence of 1µM JQ1 and/or NKs; **F-H)** Percentages of apoptotic $AnnexinV^+$ cells in NCI-H23 (**F**), NCI-H1299 (**G**), NCI-H1975 (**H**) cells co-cultured with patient-derived $CD45^+$ immune cells; **I-N)** Analysis of patient-purified NK cell activation toward NSCLC cell lines in our co-culture system. Flow cytometry analysis was applied to assess the number of activated NK cells combining IFN-γ and CD107A expression, which are enhanced in presence of BETi (**I-J**). The MFI of CD107A expression was enhanced on BETi-treated NK cells in co-cultures with NCI-H1975 compared to vehicle-treated cells (**K**). IFN-γ was measured in culture supernatants by ELISA which indicated an increased release in co-cultures with NCI-H23 and BETi (**L**, n=6 supernatants per group using cells from 3 patients, data are shown as mean ± SEM). Quantitation of $CD107A^+IFN\text{-}\gamma^+$ and $CD107^+IFN\text{-}\gamma^-$ NK cells in co-cultures with NCI-H1299 (**M,** n= 4 patients) and NCI-H1975 (**N,** n=3 patients). Data are shown as mean ± SEM. Two-side Student's t test was applied for all comparisons in this figure. ns: not significant. Source data are provided as a Source Data file.

Supplementary Figure 3

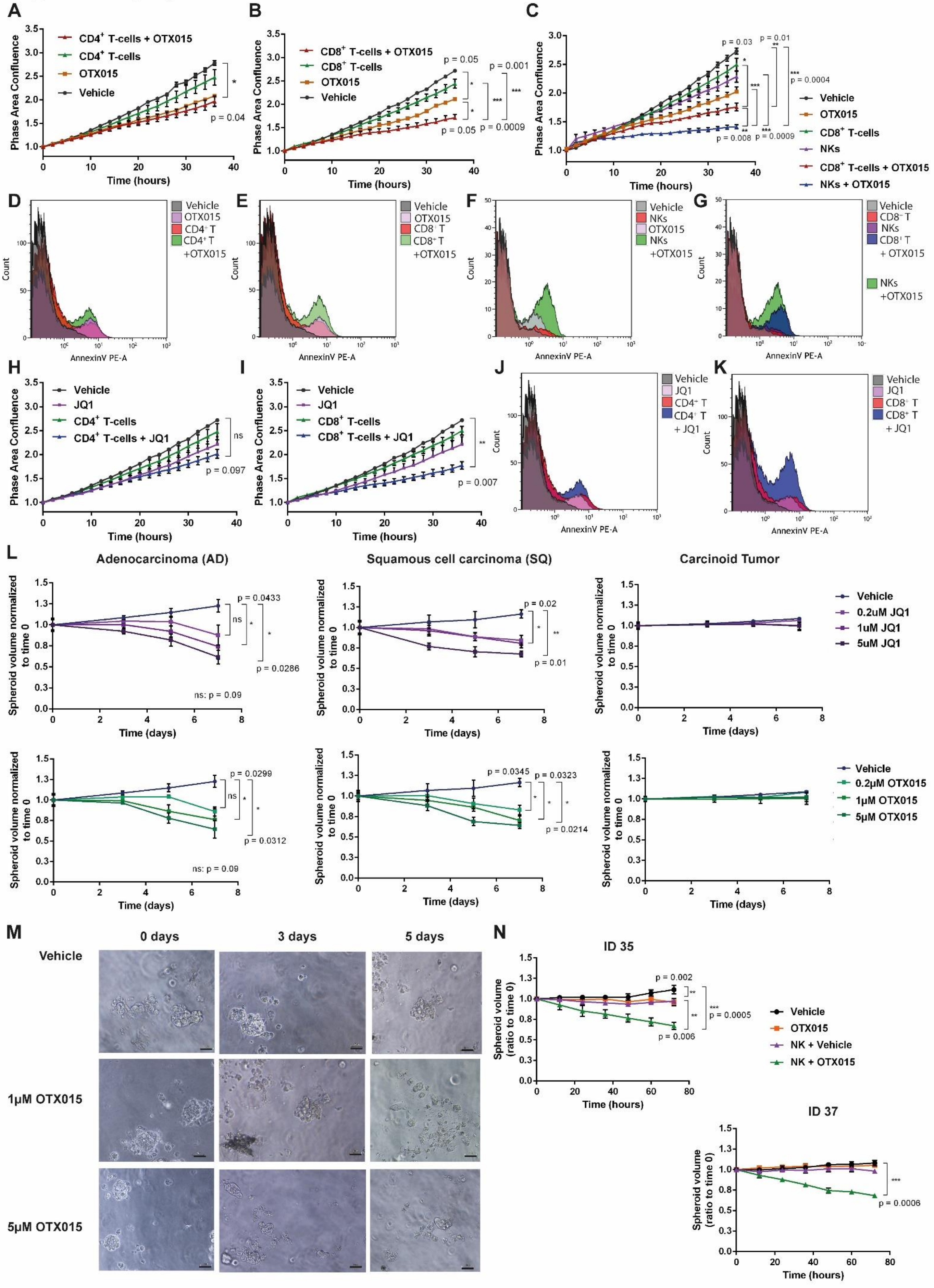

Supplementary Figure 3. A-C) Proliferation assays on NCI-H23 in co-culture with purified AD patient-derived $CD4^+$ (**A**) or $CD8^+$ T-cells (**B**) and BETi (1µM OTX015) (n=6 co-cultures per group using cells from 2 patients, data are shown as mean ± SEM). Purified NKs displayed higher anti-tumor cytotoxicity in presence of BETi compared to $CD8^+$ T cells isolated from the same patient (**C,** n=4 co-cultures per group using cells from a single patient, data are shown as mean ± SEM)). Tumor cell confluence area was measured by Incucyte S3 cell-live imaging; **D-G)** Purified $CD4^+$ T (**D**), $CD8^+$ T (**E**) or NKs (**F**) isolated from the same tumor were co-cultured with NCI-H23 and tumor apoptosis was evaluated with flow cytometry. OTX015 increased the anti-tumor efficacy of CD8 and NKs, but with higher impact on NKs (**G**); **H-I)** Proliferation assays on NCI-H23 in co-cultures with purified AD patient-derived $CD4^+$ (**H**) or $CD8^+$ T-cells (**I**) in presence of 1µM JQ1 (n=6 co-cultures per group using cells from 2 patients, data are shown as mean ± SEM); **J-K)** AnnexinV staining of tumor cells in co-cultures with $CD4^+$ (**J**) or $CD8^+$ (**K**) T cells and JQ1; **L)** Lung patient-derived CTOS were measured after BETi treatment for 7 days (n=3 patients with Adenocarcinoma-AD; n=3 patients with Squamous cell carcinoma-SQ; n=2 patients with Carcinoid Neuroendocrine tumors, data are shown as mean ± SEM). Spheroid growth was assessed through the measure of diameter and the volume was calculated; **M)** Representative images of CTOS isolated from one AD patient, *ex vivo* cultured and treated with vehicle or OTX015 (20x magnification, scale bar 40µm); **N)** Quantitation of CTOS 3D-growth in presence of BETi (1µM OTX015) or vehicle and autologous NKs from two AD patients. Spheroid volume was calculated at each time point and normalized on time 0 (n=5 spheroids per group examined for patient ID 35 or n=6 spheroids per group for patient ID 37, data are shown as mean ± SEM). Two-side Student's t test was applied for comparisons in this figure. If not specified *** $p \leq 0.001$. ns: not significant. Source data are provided as a Source Data file.

Supplementary Figure 4

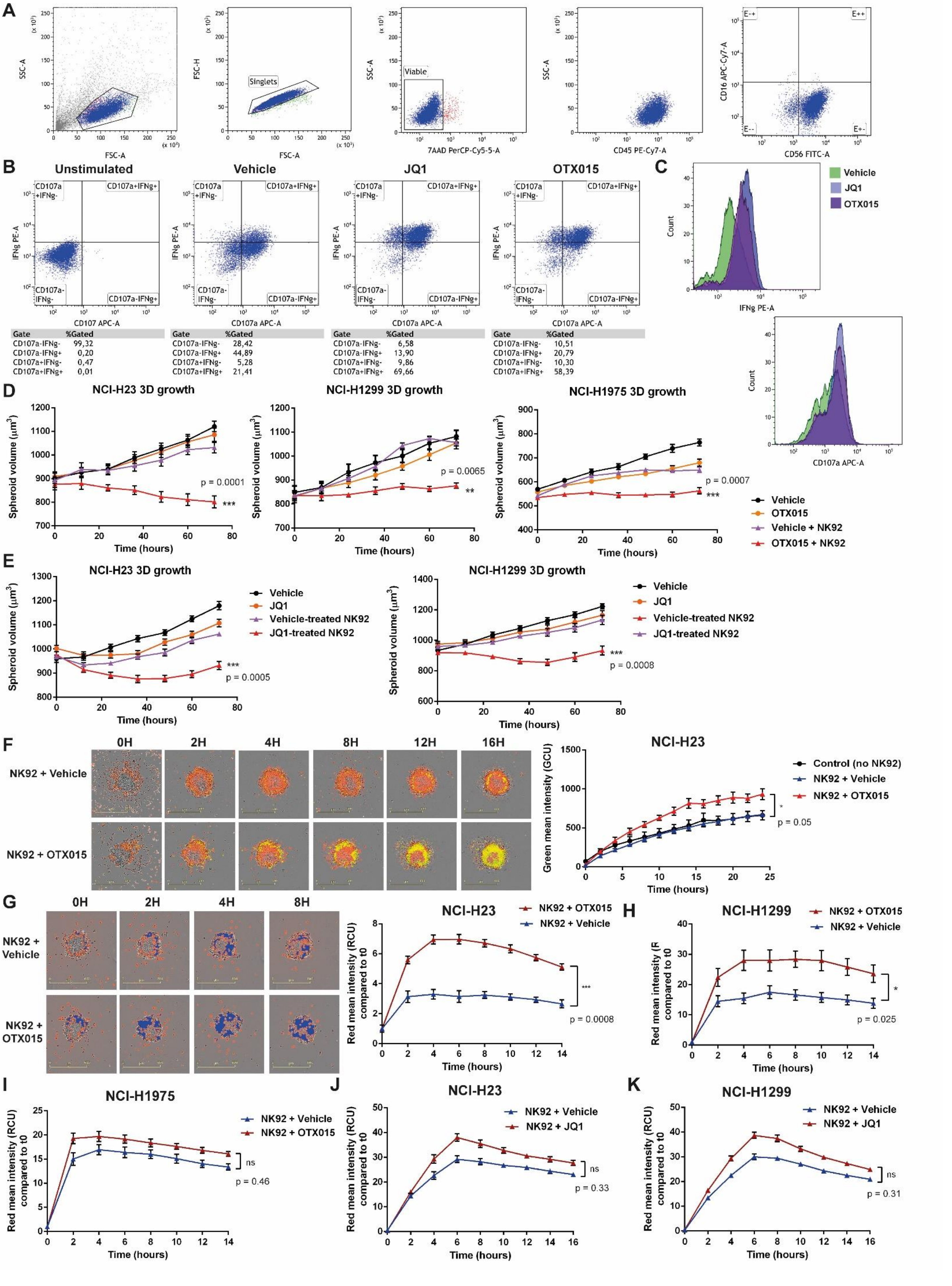

Supplementary Figure 4. A) Gating strategy and immunophenotype of NK92 by flow cytometry; **B)** Representative dot plots of IFN-γ and CD107a expression in NK92, treated or not with BETi (1µM JQ1 or OTX015), and PMA/Ionomycin stimulation for 6h. BETi increased the number of IFN-γ^+ and CD107A$^+$ NK92; **C)** Representative MFI histograms of IFN-γ (up) or CD107a (down) expression in PMA/ionomycin stimulated NK92; **D)** Spheroid 3D-growth assays on different NSCLC cell lines (NCI-H23, NCI-H1299, NCI-H1975) in co-culture with NK92 (ratio E:T 1:10) and OTX015. Spheroid volume was calculated at each time point. NKs were pre-treated with OTX015 or vehicle 24h before co-culture (n=15 spheroids per group for NCI-H23 and NCI-H1975, n=8 spheroids for NCI-H1299); **E)** Spheroid 3D growth assays on NCI-H23 and NCI-H1299 in co-culture with NK92 (ratio E:T 1:10) and JQ1 (n=12 spheroids per group for NCI-H23, n=10 for NCI-H1299); **F)** Tumor spheroids of NCI-H23 cells were co-cultured with red-stained NK92 and OTX015. IncuCyte® Cytotox Green Reagent was added to the media to label cytotoxic cells. Representative brightfield images with red and green fluorescent signals were analysed by Incucyte software (4x magnification, scale bar 600µm). Green Fluorescence mean intensity (GCU) was calculated after normalization with basal fluorescence at time 0 in each sample, unveiling an increased tumor cell cytotoxicity in presence of OTX015 (n=6 spheroids per group); **G)** Immunological synapse formation between red-stained NK92 and NCI-H23 spheroids was assessed by quantifying RCU within the brightfield area of each spheroid (4x magnification, scale bar 600µm). Blue masks correspond to analysed areas. NK92 were pre-treated with OTX015 for 24h before co-cultures and displayed an increased immunological synapse formation (n=3 independent experiments); **H-I)** Quantitation of immunological synapses with NCI-H1299 (**H**) or NCI-H1975 (**I**) in case of OTX015 pre-treatment of NK92 (n=5 spheroids per group); **J-K)** Quantitation of immunological synapses with NCI-H23 (**J**) or NCI-H1299 (**K**) in case of JQ1 pre-treatment of NK92 (n=11 spheroids per group). All data are shown as mean ± SEM. Two-side Student's t test was applied for comparisons in this figure. ns: not significant. Source data are provided as a Source Data file.

Supplementary Figure 5

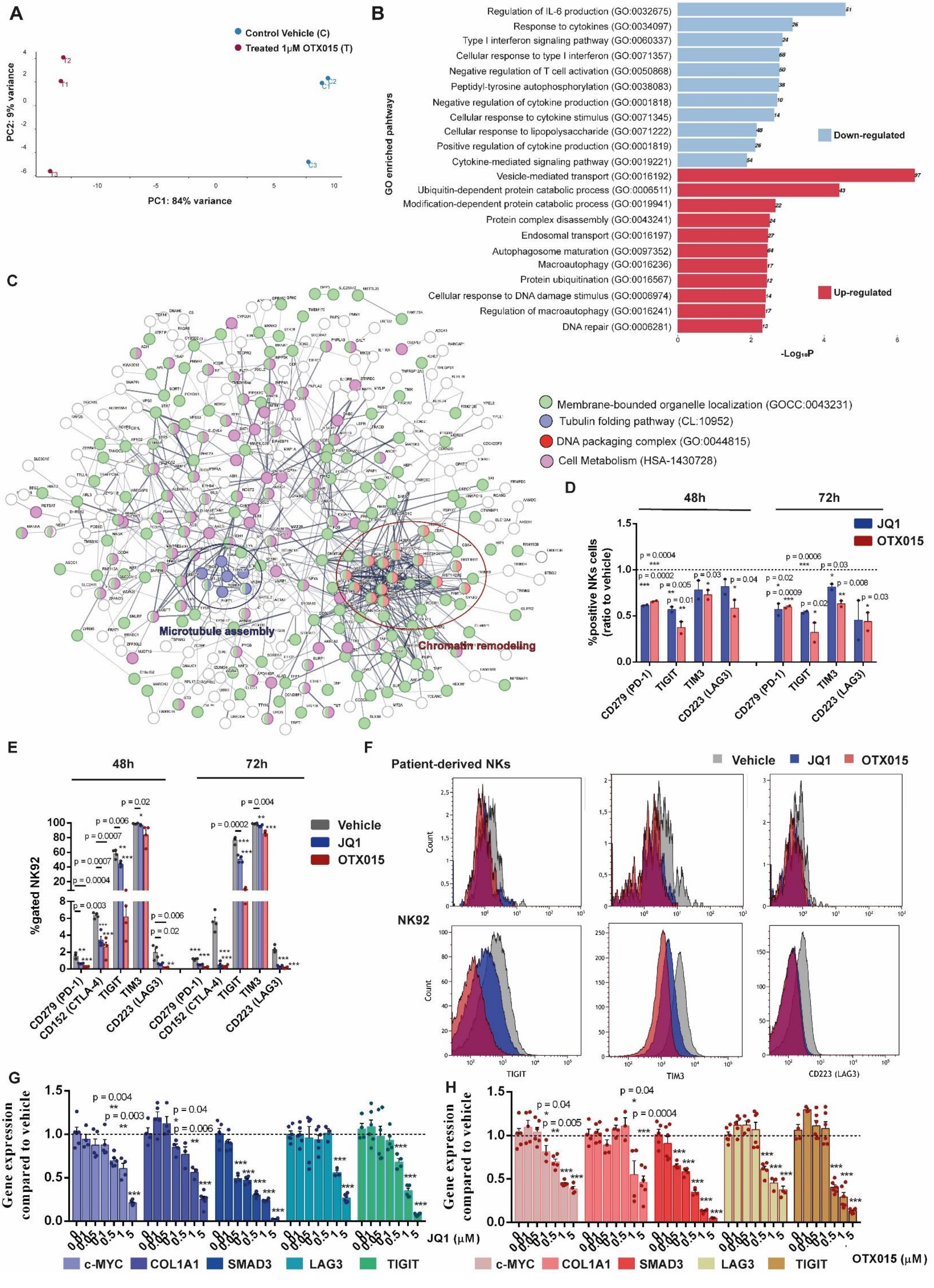

Supplementary Figure 5. A) Principal Component Analysis (PCA) of RNA-sequencing (RNA-seq) comparing NK92 treated with 1µM OTX015 for 24h (T_1-T_2-T_3) with vehicle (C_1-C_2-C_3) in three independent replicates; **B)** Gene ontology (GO) enrichment of RNA-seq down- (*blue*) and up- (*red*) regulated genes, indicating that several immune-related pathways were affected by OTX015 treatment; **C)** STRING Network analysis of top-scoring up-regulated genes from the RNA-seq analysis ($Log_2FC>0.7$). Genes belonging to the main enriched GO pathways are highlighted. Most of the genes encode for intracellular organelle membrane proteins and are involved in vesicle trafficking, chromatin remodelling, or cell metabolism; **D-F)** Flow cytometry analysis of IC molecule expression on the surface of patient-derived intratumor NKs (**D**, n=2 patients including 1 AD and 1 SQ, data are shown as mean ± SEM) and NK92 cells (**E**, n=4 independent experiments, data are shown as mean ± SEM) treated with BETi (1µM JQ1 or OTX015) for 48h or 72h. The results indicated a significant reduction of positive IC^+ cells induced by BETi (**D-E**) and an impairment of the MFI of TIM3, LAG3, and TIGIT expression (**F**); **G-H)** Dose-response analysis of JQ1 (**G**) and OTX015 (**H**) in NK92 cells to assess BRD4 target inhibition after 24h. qPCR was used to measure transcript down-regulation following BETi treatment. Values were normalized for housekeeping gene expression and expressed as fold change (n=6 biological independent samples, data are shown as mean ± SEM). Two-side Student's t test was applied for the comparisons in this figure. If not specified *** $p \leq 0.001$. Source data are provided as a Source Data file.

Supplementary Figure 6

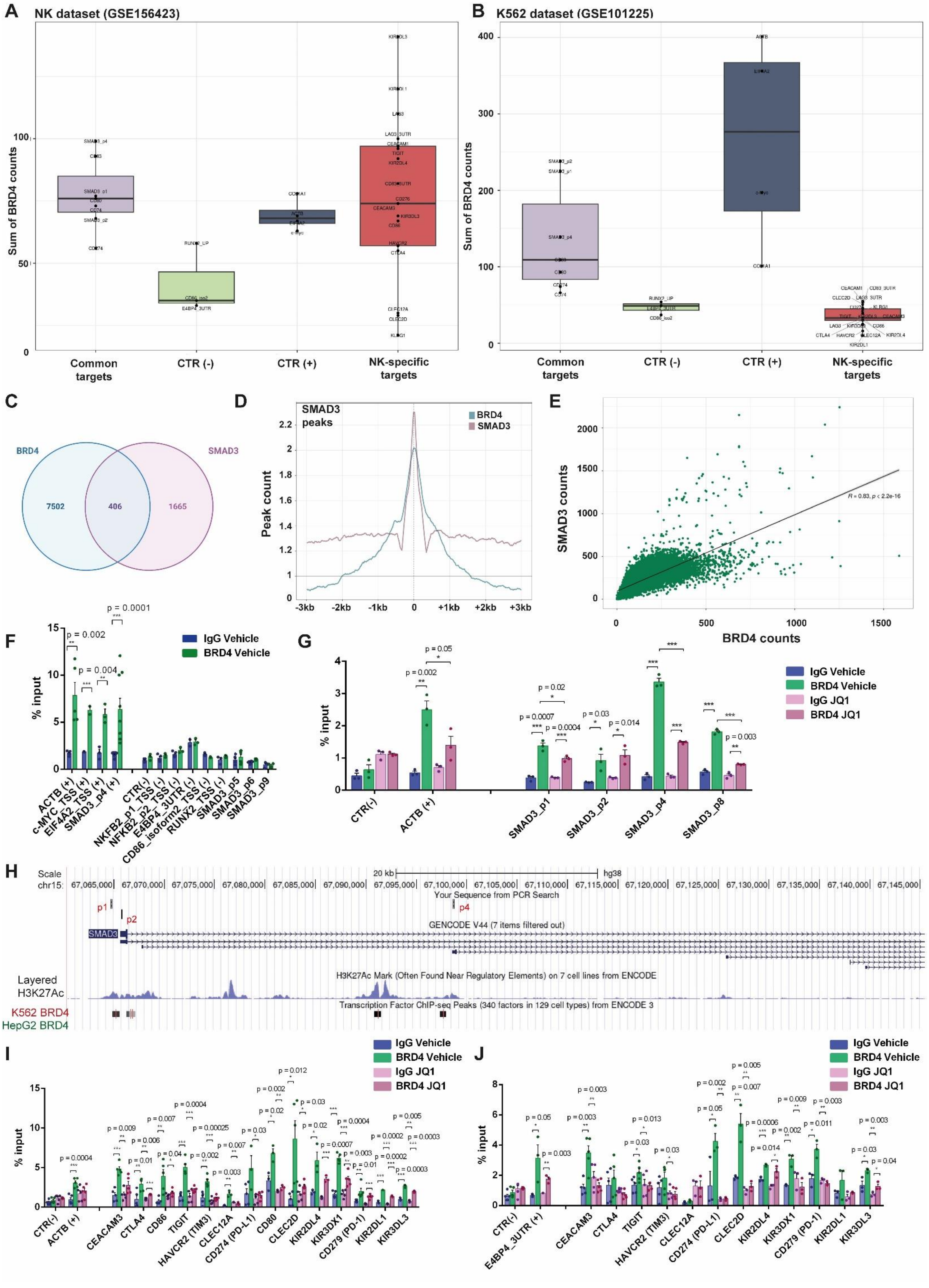

Supplementary Figure 6. **A-B)** Box plots illustrating the BRD4 counts for our ChIP targets in ChIP-seq public datasets available for patient-derived NKs (GSE156423[29]) **A**) and K562 (GSE101225[30], **B**). Common targets included genes bound by BRD4 in both cell contexts (*grey boxes*), whereas NK-specific targets were detected to be recognized by BRD4 in NKs only (*red boxes*). Positive (*blue boxes*) and negative (*green boxes*) control regions were taken from our ChIP controls in NK92; **C)** Venn diagram illustrating the overlap between BRD4 (GSE101225[30]) and SMAD3 (GSE231137[30]) peaks in K562 cells; **D)** Metaprofiles of BRD4 and SMAD3 on SMAD3 peaks; **E)** Scatter plot showing the correlation between BRD4 and SMAD3 counts on TSS of coding genes; **F)** Negative (-) and positive (+) controls for ChIP analysis in NK92 cells using the anti-BRD4 antibody. Values are expressed as % of input (n=5 biological independent samples, data are shown as mean ± SEM); **G)** Treatment with 1µM JQ1 for 48h induced BRD4 detachment from SMAD3 promoter regions in NK92 cells, as detected by ChIP (n=3 independent experiments, data are shown as mean ± SEM); **H)** Illustration of the genomic region encoding for SMAD3 transcripts. The layered H3K27Ac track is relative to K562 cells. Upper highlighted sequences (P1, P2, P4) indicate the amplified regions from our BRD4 ChIP experiments. The bottom tracks are related to BRD4 ChIP-seq peaks from ENCODE project in K562 or HepG2 cells. The image was modified from Genome Browser; **I-J)** ChIP analysis of BRD4 (**I**) or SMAD3 (**J**) in NK92 cells treated with 1µM JQ1 for 48h identified its detaching from target regulatory regions (n=5 biological independent samples, data are shown as mean ± SEM). Two-side Student's t test was applied for the comparisons in this figure. If not specified *** p≤0.001. Source data are provided as a Source Data file.

Supplementary Figure 7

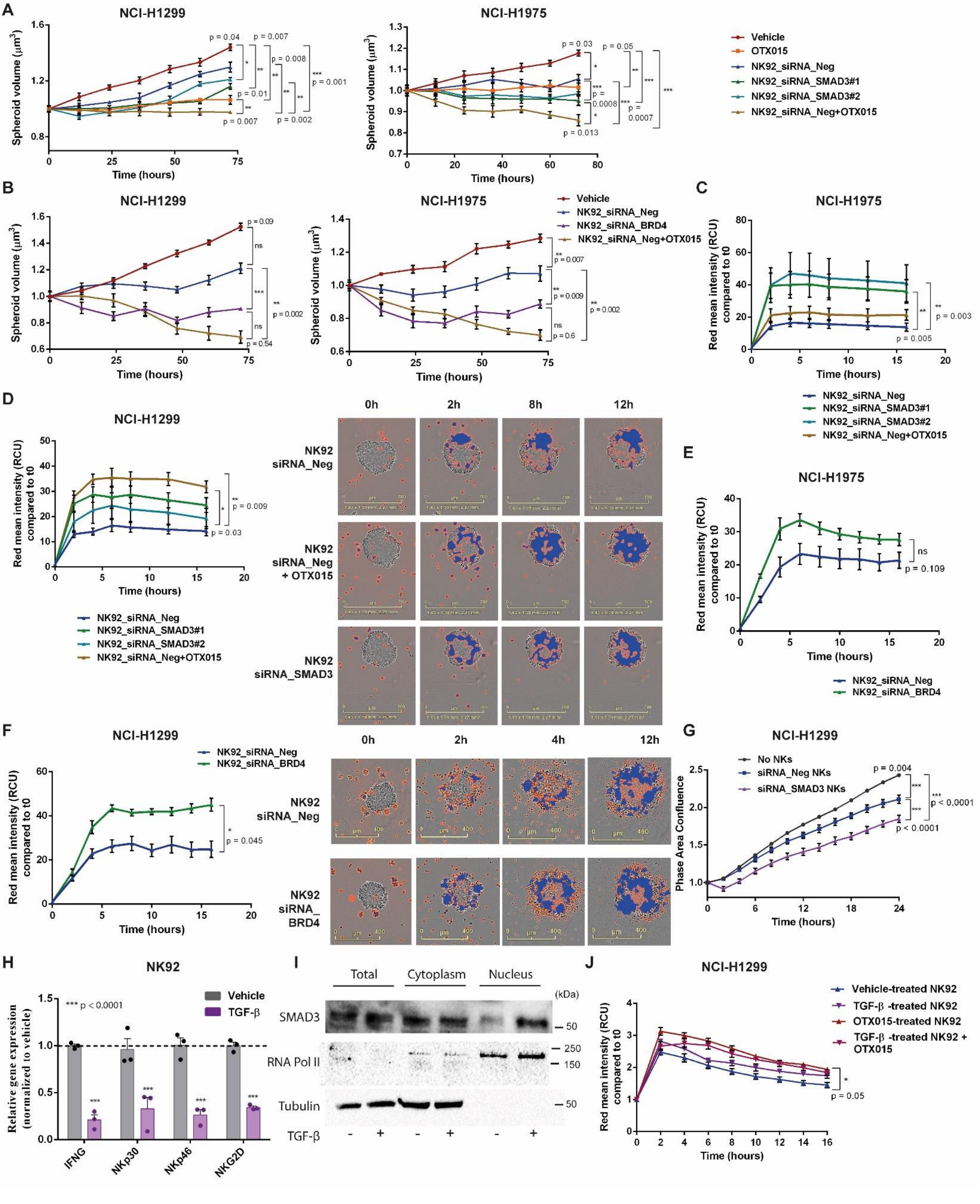

Supplementary Figure 7. A) Spheroid 3D-growth assays on NCI-H1299 or NCI-H1975 spheroids in co-culture with red-stained $SMAD3^{KD}$ NK92 cells or siRNA-CTR NK92 cells. Spheroid volume was calculated at each time point. NK cells were pre-treated with OTX015 or vehicle 24h before and during co-culture (n=6 spheroids per group); **B)** Spheroid 3D-growth assays on NCI-H1299 or NCI-H1975 spheroids in co-culture with red-stained $BRD4^{KD}$ NK92 cells or siRNA-CTR NK92 cells (n=6 spheroids per group, data are shown as mean ± SEM); **C-D)** Immunological synapse formation between red-stained $SMAD3^{KD}$ or siRNA-CTR NK92 and NCI-H1975 (**C**) or NCI-H1299 (**D**) spheroids was assessed by quantifying Red Fluorescence mean intensity (RCU) within the brightfield area of each spheroid by Incucyte 2020B software (4x magnification, scale bar 600μm). Blue masks correspond to quantified areas (n=4 spheroids per group, data are shown as mean ± SEM); **E-F)** Immunological synapse formation between red-stained $BRD4^{KD}$ or siRNA-CTR NK92 and NCI-H1975 (**E**) or NCI-H1299 (**F**) spheroids (4x magnification, scale bar 400μm). Blue masks correspond to quantified red fluorescent areas (n=6 spheroids per group, data are shown as mean ± SEM); **G)** The KD of SMAD3 in primary NKs increased their anti-tumor activity toward NCI-H1299 cells (n=4 co-cultures using cells from a single patient, data are shown as mean ± SEM); **H)** TGF-β administration (5ng/ml for 6h) induced a rapid transcriptional down-regulation of known TGF-β down-stream targets in NK cells, as detected by qPCR (n=3 independent experiments, data are shown as mean ± SEM); **I)** Assessment of increased SMAD3 nuclear translocation after TGF-β administration in NK92 cells. The quality of nuclear/cytoplasm fractions was assessed by RNA-PolII expression in the cell nucleus and α-tubulin for cell cytoplasm. Total cell lysates were loaded as controls; **J)** Immunological synapse formation between red-stained NK92 and NCI-H1299 spheroids (n=6 spheroids per group, data are shown as mean ± SEM). NK92 were pre-treated for 16h with TGF-β (5ng/ml) or OTX015 (1μM) or both before starting the co-cultures. Two-side Student's t test was applied for the comparisons in this figure. If not specified *** $p \leq 0.001$. ns: not significant. Source data are provided as a Source Data file.

Supplementary Figure 8

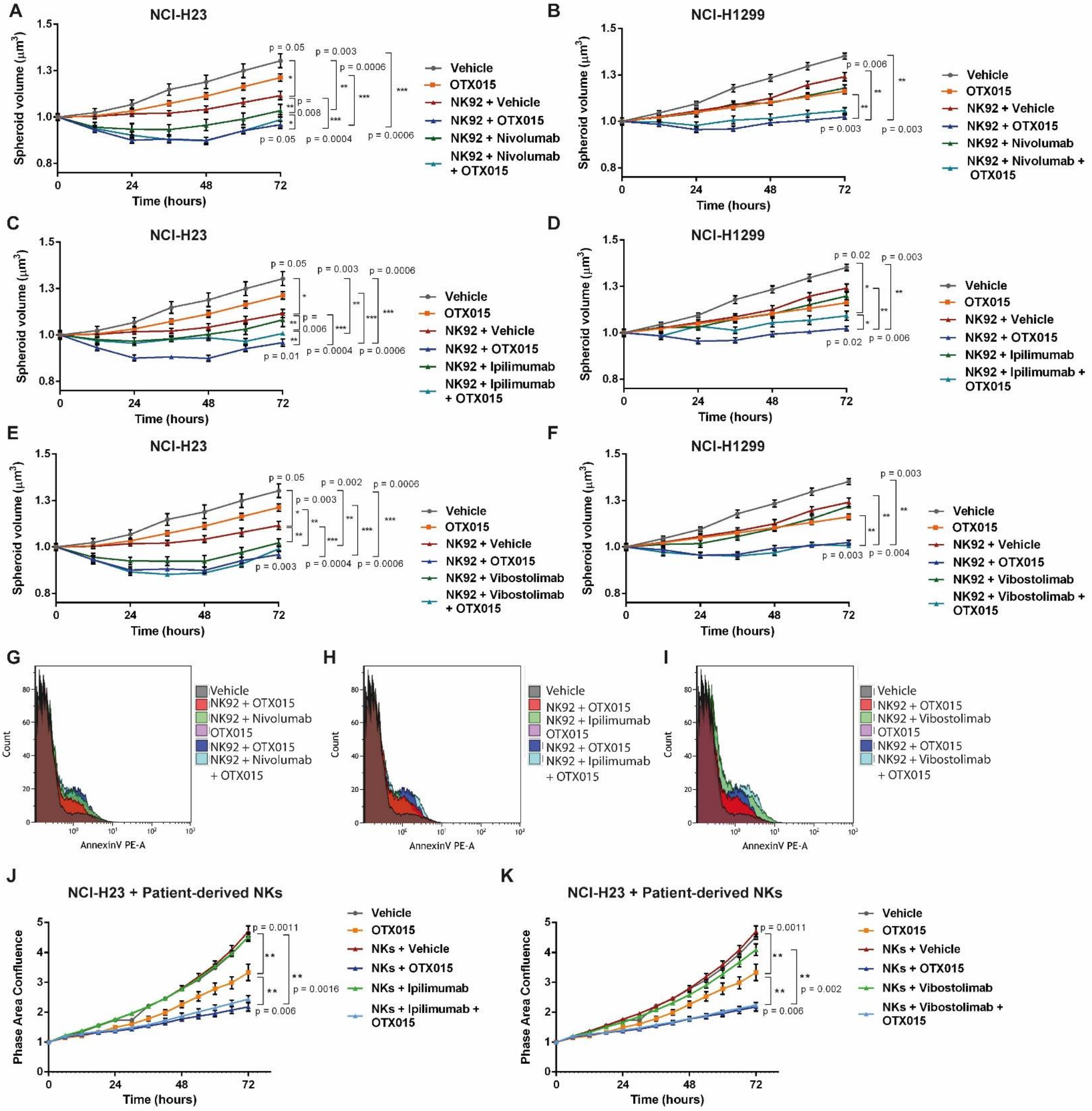

Supplementary Figure 8. A-F) Spheroid 3D growth assays on NCI-H23 and NCI-H1299 spheroids in co-culture with red-stained NK92 cells in presence of 1μM OTX015 and/or IC inhibitors: anti-PD1 Nivolumab 15μg/ml (**A-B**), anti-CTLA4 Ipilimumab 5μg/ml (**C-D**) or anti-TIGIT Vibostolimab 10μg/ml (**E-F**) (n=12 spheroids per group for NCI-H23, n=11 spheroids per group for NCI-H1299, data are shown as mean ± SEM); **G-I)** Quantitation of tumor apoptosis (NCI-H23) by AnnexinV staining in co-cultures with NK92 cells in presence of OTX015, IC inhibitors or their combination; **J-K)** Representative proliferation assays using co-cultures of patient-derived NKs and NCI-H23 cells. The efficacy of BETi (1μM OTX015) in promoting NK cytotoxicity toward tumor cells was compared with distinct IC inhibitors, anti-CTLA4 Ipilimumab (5μg/ml, **J**) or anti-TIGIT Vibostolimab (10μg/ml, **K**). Tumor cell confluence was assessed with Incucyte 2020B Software (n=3 co-cultures using cells from a single patient, data are shown as mean ± SEM). Two-side Student's t test was applied for the comparisons for this figure. Source data are provided as a Source Data file.

Supplementary Figure 9

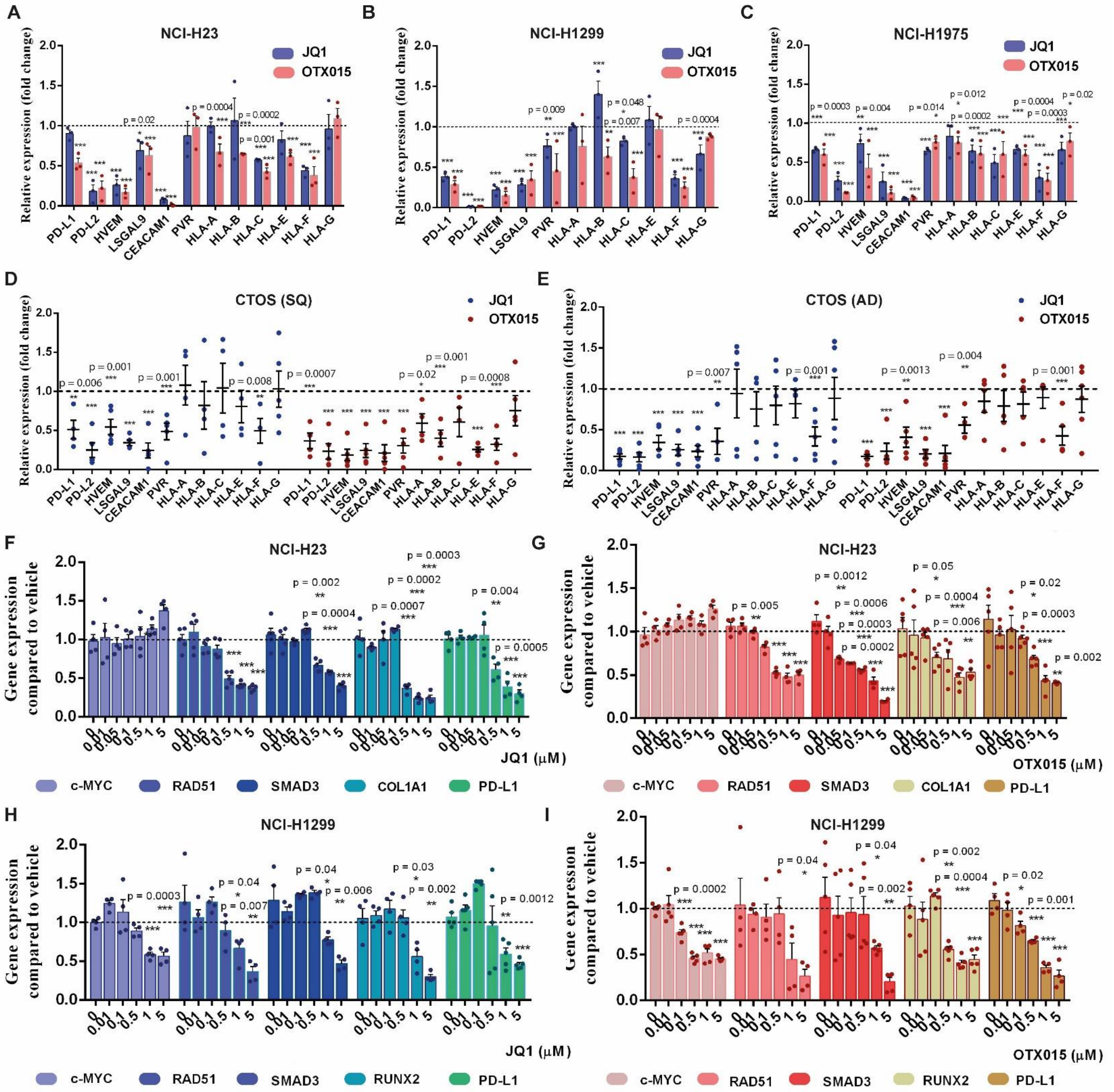

Supplementary Figure 9. A-C) IC ligands and HLA class I molecule expression in BETi-treated NSCLC cell lines by qPCR. Cells were treated with 1μM JQ1 or OTX015 for 24h. Values were normalized for housekeeping gene expression and expressed as fold change (n=3 independent experiments, data are shown as mean ± SEM); **D-E)** BETi treatment (1μM JQ1 or OTX015) impaired the expression of ICs and HLAs in NSCLC-patient derived CTOS (**D**, n=5 patients with SQ patients; **E**, n=6 patients with AD patients, data are shown as mean ± SEM); **F-G)** Dose-response analysis of JQ1 (**F**) and OTX015 (**G**) in NCI-H23 cells to assess BRD4 target inhibition after 24h. qPCR was used to measure transcript down-regulation following BETi treatment. Values were normalized for housekeeping gene expression and expressed as fold change (n=5 biological independent samples, data are shown as mean ± SEM); **H-I)** Dose-response analysis in NCI-H1299 cells using JQ1 (**H**) or OTX015 (**I**) (n=5 biological independent samples, data are shown as mean ± SEM). Two-side Student's t test was applied for the comparisons in this figure. If not specified *** p≤0.001. Source data are provided as a Source Data file.

Supplementary Table 1. List of fluorophore-conjugated antibodies used for flow cytometry analyses.

Target	Fluorophore	Catalog#	Company	Amount (µl for 1x10^6 cells)
hCD45	FITC	342408	BD	0.5
hCD45	PE-Cy™7	557748	BD	0.5
hCD3	FITC	300406	Biolegend	1
hCD4	APC	130-113-784	Miltenyi Biotech	1
hCD8	APC-Vio®770	130-110-819	Miltenyi Biotech	1
hCD14	PE	342408	BD	3
hCD19	PE-Vio® 770	130-113-170	Miltenyi Biotech	1
hCD56	FITC	562794	BD	3
hCD16	APC-H7	560195	BD	2
hCD94	APC	559876	BD	3
hIFN-γ	PE	340452	BD	5
hCD107a	APC	130-119-869	Miltenyi Biotech	3
AnnexinV	PE	559763	BD	5
hCD152 (CTLA4)	PE	130-118-357	Miltenyi Biotech	3
hCD279 (PD1)	APC	130-117-694	Miltenyi Biotech	1
hTIGIT	PE	130-116-814	Miltenyi Biotech	1
hTIM3	APC	130-120-700	Miltenyi Biotech	1
hCD223	APC-Vio®770	130-130-284	Miltenyi Biotech	3
mCD45	PE-Vio® 770	130-117-529	Miltenyi Biotech	1
mCD4	FITC	553729	BD	1
mCD8	APC-Cy7	557654	BD	1
mCD19	APC	17-0193-80	Thermo Fisher	1
mNK1.1	APC	17-5941-81	Thermo Fisher	1
mCD335 (NKp46)	APC	130-117-514	Miltenyi Biotech	1
mCD279 (PD1)	APC-Cy7	135223	Biolegend	2
mTIGIT	PE	130-120-296	Miltenyi Biotech	2
mCD152 (CTLA4)	PE	130-102-570	Miltenyi Biotech	2
mCD107a	FITC	130-102-191	Miltenyi Biotech	3

Uncropped blots

Fig.3f

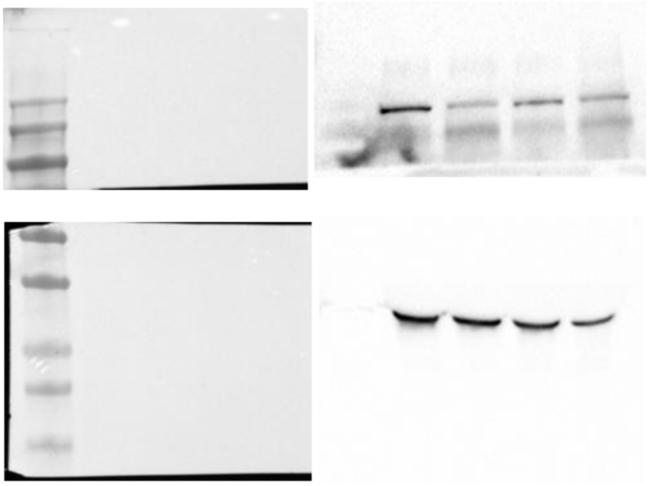

BRD4

Actin

Fig.3k

SMAD3

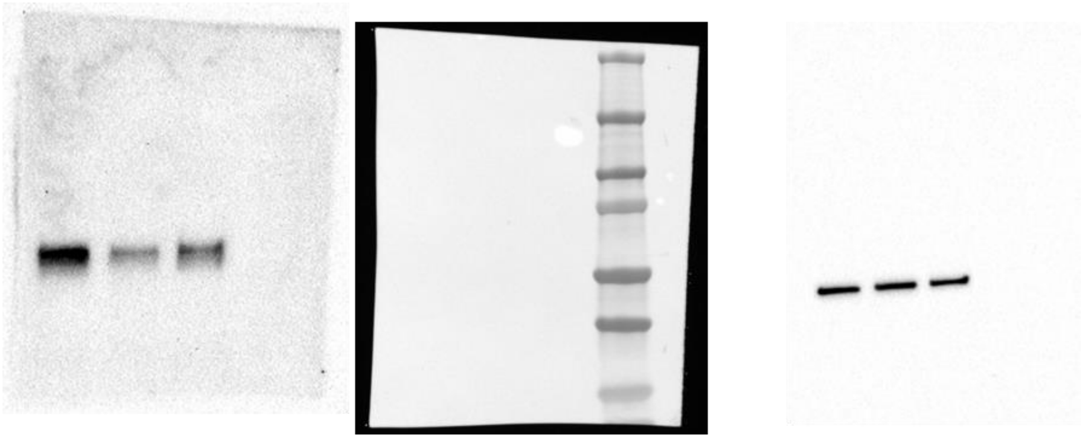

Actin

Fig. 3m

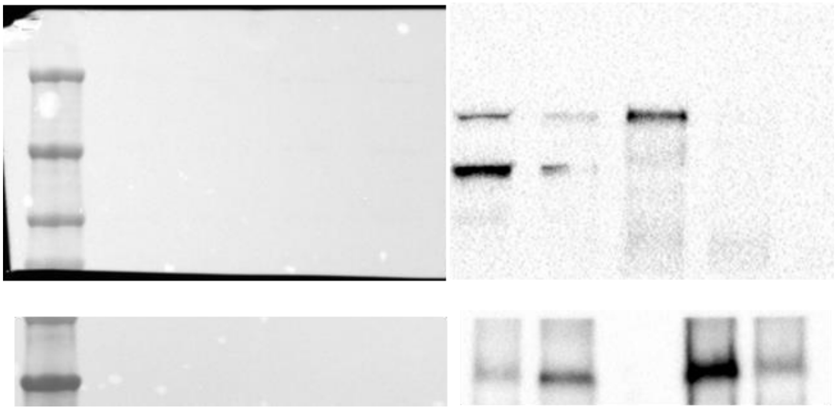

BRD4

SMAD3

Fig. 4c

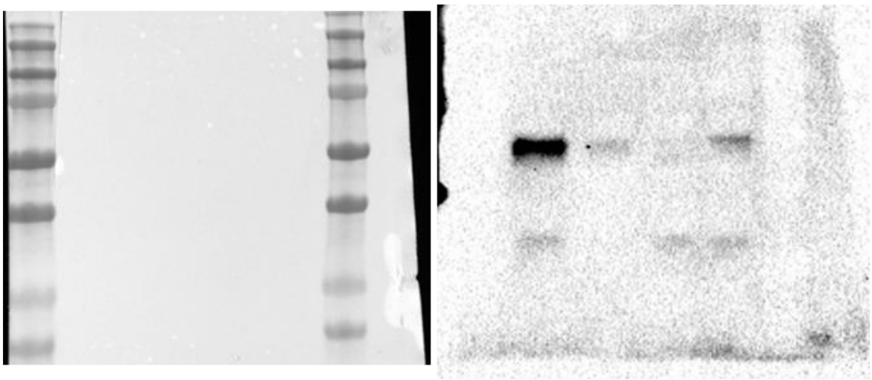

SMAD3

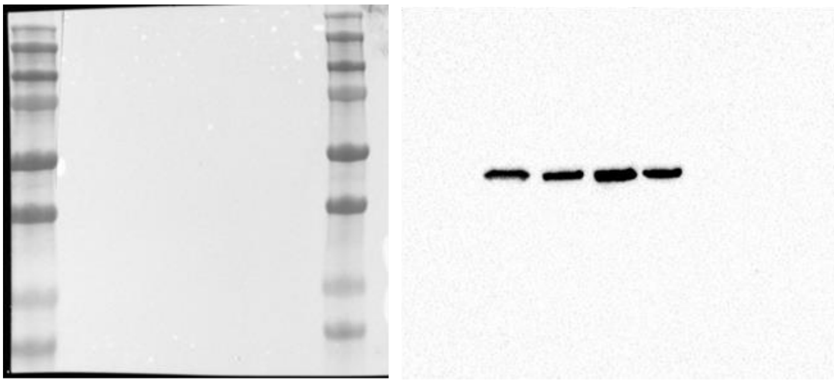

Actin

SMAD3

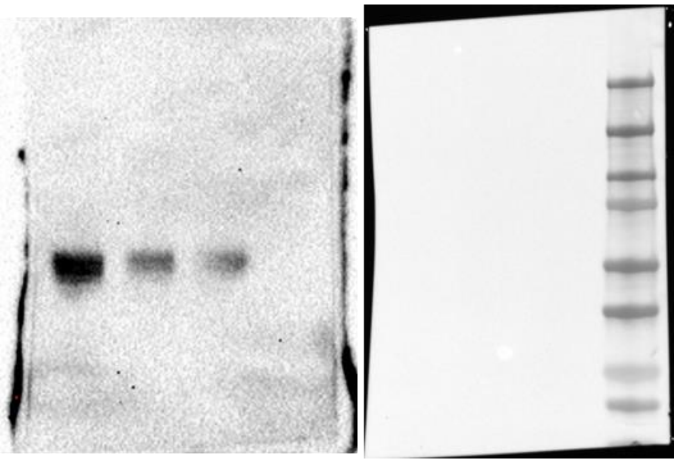

Actin

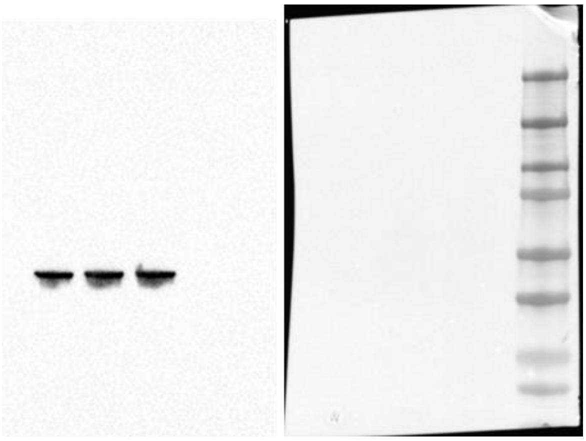

Fig. 4h

SMAD3

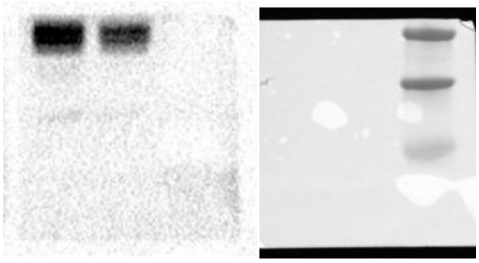

Actin

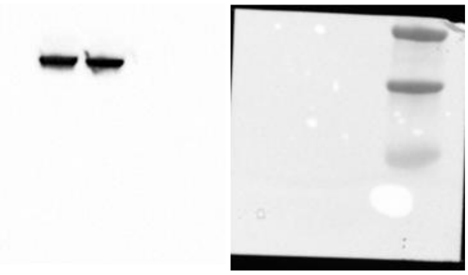

Suppl. Fig. 7i

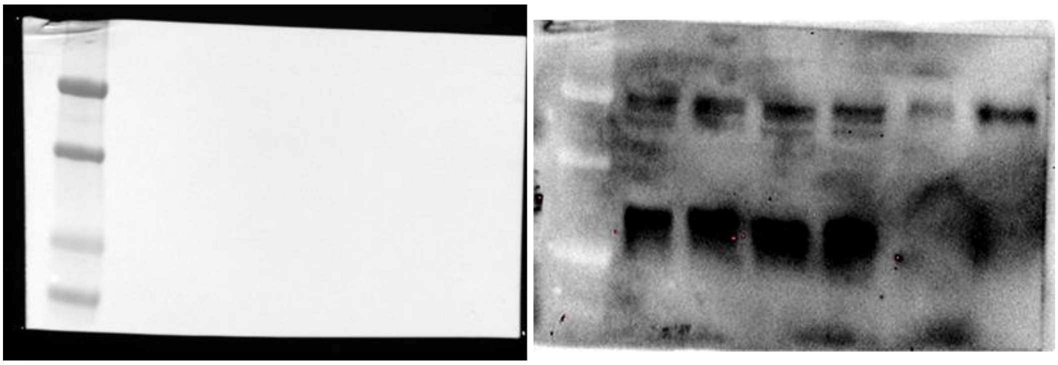

BRD4

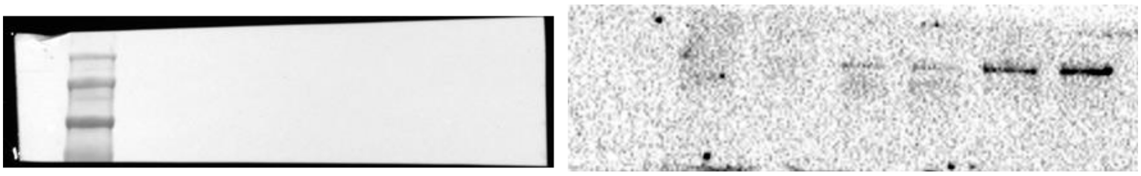

RNA Pol II

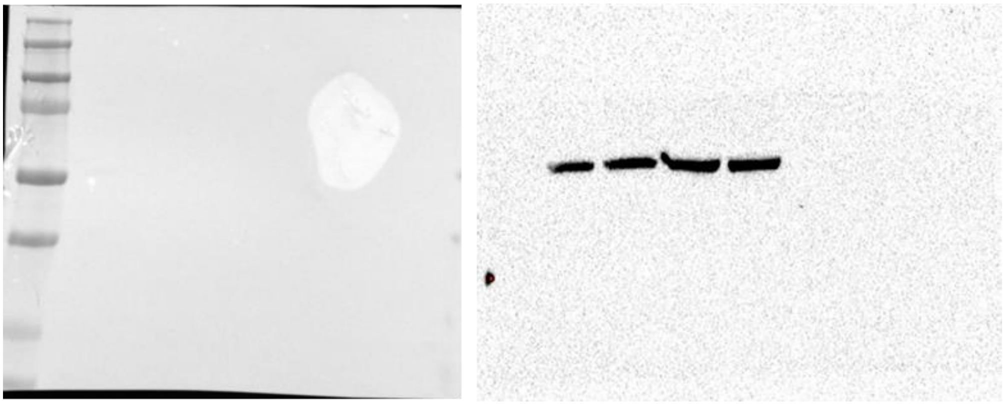

Tubulin
