# Supplementary material for: BET inhibitors drive Natural Killer activation in non-small cell lung cancer via BRD4 and SMAD3
Source: Nat Commun. 2024 Mar 22;15:2567. doi: 10.1038/s41467-024-46778-8 (PMC10960013; doi:10.1038/s41467-024-46778-8)
Supplement: Supplementary file 2 — Description of Additional Supplementary Files [file 41467_2024_46778_MOESM2_ESM.pdf]

Title: Supplementary Data 1.
Description: Table with the clinical-pathological features of lung cancer patients included in the study.

Title: Supplementary Data 2.
Description: Table with the list of primer sequences applied in qPCR analyses.

Title: Supplementary Data 3.
Description: Table with the list of primer sequences applied in ChIP analyses.

Title: Supplementary video 1.
Description: Ex vivo co-cultures of NSCLC cells (NCI-H23) and patient-derived TILs (green-stained) in presence of vehicle or BETi (OTX015). Acquired with Incucyte system (4x magnification, scale bar 400µm).

Title: Supplementary video 2.
Description: Ex vivo co-cultures of AD patient-derived CTOS and autologous purified NK cells (red-stained) in presence of vehicle or BETi (OTX015). Acquired with Incucyte system (4x magnification, scale bar 300µm).

Title: Supplementary video 3.
Description: Co-cultures between NSCLC cells (NCI-H1299) and NK92 cells (green-stained) in presence of vehicle or BETi (OTX015). Acquired with Incucyte system (4x magnification, scale bar 400µm).

Title: Supplementary video 4.
Description: Co-cultures between NSCLC 3D spheroids (NCI-H1299) and NK92 cells (red-stained) in presence of vehicle or BETi (OTX015). Acquired with Incucyte system (4x magnification, scale bar 700µm).
