# Supplementary material for: BET inhibitors drive Natural Killer activation in non-small cell lung cancer via BRD4 and SMAD3
Source: Nat Commun. 2024 Mar 22;15:2567. doi: 10.1038/s41467-024-46778-8 (PMC10960013; doi:10.1038/s41467-024-46778-8)
Supplement: Supplementary file 3 — Reporting Summary [file 41467_2024_46778_MOESM3_ESM.pdf]

nature portfolio

Corresponding author(s): Francesca Reggiani
Valentina Sancisi

Last updated by author(s): Feb 8, 2024

Reporting Summary

Nature Portfolio wishes to improve the reproducibility of the work that we publish. This form provides structure for consistency and transparency in reporting. For further information on Nature Portfolio policies, see our Editorial Policies and the Editorial Policy Checklist.

Statistics

For all statistical analyses, confirm that the following items are present in the figure legend, table legend, main text, or Methods section.

n/a	Confirmed	
[ ]	[x]	The exact sample size (*n*) for each experimental group/condition, given as a discrete number and unit of measurement
[ ]	[x]	A statement on whether measurements were taken from distinct samples or whether the same sample was measured repeatedly
[ ]	[x]	The statistical test(s) used AND whether they are one- or two-sided<br>*Only common tests should be described solely by name; describe more complex techniques in the Methods section.*
[x]	[ ]	A description of all covariates tested
[ ]	[x]	A description of any assumptions or corrections, such as tests of normality and adjustment for multiple comparisons
[ ]	[x]	A full description of the statistical parameters including central tendency (e.g. means) or other basic estimates (e.g. regression coefficient) AND variation (e.g. standard deviation) or associated estimates of uncertainty (e.g. confidence intervals)
[ ]	[x]	For null hypothesis testing, the test statistic (e.g. *F*, *t*, *r*) with confidence intervals, effect sizes, degrees of freedom and *P* value noted<br>*Give P values as exact values whenever suitable.*
[x]	[ ]	For Bayesian analysis, information on the choice of priors and Markov chain Monte Carlo settings
[x]	[ ]	For hierarchical and complex designs, identification of the appropriate level for tests and full reporting of outcomes
[x]	[ ]	Estimates of effect sizes (e.g. Cohen's *d*, Pearson's *r*), indicating how they were calculated

Our web collection on statistics for biologists contains articles on many of the points above.

Software and code

Policy information about availability of computer code

Data collection	Incucyte v 2022B Software (Sartorius), Bio-Rad CFX Manager v 3.1 Software (Biorad), Image Lab v 5.2 Software (Biorad), BD FACSDIVA v 8 Software (BD), Living Image Software (PerkinElmer), sra tools (source GitHub).
Data analysis	Incucyte v 2022B Software (Sartorius), FastQC v 0.11.8 Software (source www.bioinformatics.babraham.ac.uk/projects/fastqc/), STAR v 2.7 algorithm (source GitHub), RSEM algorithm v.1.3.1 (source GitHub), R software v 4.1.0 (https://cran.r-project.org/bin/windows/base/), STRING v 11.5 (http://www.string-db.org/), FIMO algorithm (source MEME suit), ImageJ software v 1.54 (source https://imagej.nih.gov/ij/), deepTools (https://deeptools.readthedocs.io/en/develop/index.html), bedtools (source GitHub), GraphPad Prism v 9.3.0 Software (GraphPad), G*Power v 3.1.9.7 (http://www.gpower.hhu.de), Kaluza v 2.1 Software (Beckman Coulter).

For manuscripts utilizing custom algorithms or software that are central to the research but not yet described in published literature, software must be made available to editors and reviewers. We strongly encourage code deposition in a community repository (e.g. GitHub). See the Nature Portfolio guidelines for submitting code & software for further information.

Data

Policy information about availability of data

All manuscripts must include a data availability statement. This statement should provide the following information, where applicable:

- Accession codes, unique identifiers, or web links for publicly available datasets
- A description of any restrictions on data availability
- For clinical datasets or third party data, please ensure that the statement adheres to our policy

The RNA-seq data generated in this study have been deposited in the ArrayExpress database under the accession code E-MTAB-12853 and in the ENA database under the accession number ERP146384 (https://www.ebi.ac.uk/ena/browser/view/PRJEB61285). The following ChIP-seq public datasets were analysed: GSE156423 (https://www.ncbi.nlm.nih.gov/geo/query/acc.cgi?acc=GSM4730573) ChIP-seq for BRD4 in NKs isolated from peripheral blood of two healthy donors (REF. 29; 10.3389/fimmu.2021.626255), GSE101225 (ENCODE project n°ENCSR583ACG; https://www.encodeproject.org/experiments/ENCSR583ACG/) ChIP-seq for BRD4 in K562 cell line, GSE231137 (ENCODE project n°ENCSR140GLO; https://www.encodeproject.org/experiments/ENCSR140GLO/) ChIP-seq for SMAD3 in K562 cell line (REF. 30; DOI 10.1126/science.1105136). The remaining data are available within the Article, Supplementary Information or Source Data file. Source data are provided with this paper.All data supporting the findings of this study are available within the paper and its Supplementary Information.

Research involving human participants, their data, or biological material

Policy information about studies with human participants or human data. See also policy information about sex, gender (identity/presentation), and sexual orientation and race, ethnicity and racism.

Reporting on sex and gender	No sex- or gender-based analysis was performed. This did not apply to the study.
Reporting on race, ethnicity, or other socially relevant groupings	No information about race, ethnicity or other socially relevant grouping was collected. This did not apply to the study.
Population characteristics	53 patients with a lung cancer diagnosis and undergoing surgery at the Azienda USL-IRCCS of Reggio Emilia (Italy) from 2018 to 2023. Both sexes were included, age were in the 51-84 range with average 70.
Recruitment	Consecutive patients were recruited for the study and during the revision process. Patients with metastatic diseases were a priori excluded from enrollment. Patients undergoing surgery for lung cancer were proposed to adhere to this study and a signed informed consensus was collected from each patient. We are not aware of any bias that may impact the results of the study.
Ethics oversight	Comitato Etico dell'Area Vasta Emilia Nord (AVEN) - Reggio Emilia district (Authorization code 196/2017).

Note that full information on the approval of the study protocol must also be provided in the manuscript.

Field-specific reporting

Please select the one below that is the best fit for your research. If you are not sure, read the appropriate sections before making your selection.

[x] Life sciences [ ] Behavioural & social sciences [ ] Ecological, evolutionary & environmental sciences

For a reference copy of the document with all sections, see nature.com/documents/nr-reporting-summary-flat.pdf

Life sciences study design

All studies must disclose on these points even when the disclosure is negative.

Sample size	For mouse studies, assuming a coefficient of variation (CV) of tumor growth equal for each arm, at least 5 mice per arm were necessary to detect, with 0.8 potency, a halving of tumor growth (alpha error equal to 0.05). For analyses on human specimens, sample size was determined by samples availability. All samples resulting quantitatively and qualitatively suitable for the required analyses were included in the study.
Data exclusions	For mouse studies, mice were excluded if no tumor bioluminescence was detected (absence of tumor engraftment) or if mice died before the experimental endpoints.
Replication	Each experiment was replicated multiple times (>3 up to 6).
Randomization	For mouse studies, when a basal bioluminescence signal (corresponding to tumor engraftment) was detected, mice were randomized into four experimental groups, before starting treatments. For analyses on human specimens, no randomization was applied. For this reason, covariates such as age were not relevant.
Blinding	Blinding was not applicable to this study since no patients randomization was applied.

Reporting for specific materials, systems and methods

We require information from authors about some types of materials, experimental systems and methods used in many studies. Here, indicate whether each material, system or method listed is relevant to your study. If you are not sure if a list item applies to your research, read the appropriate section before selecting a response.

Materials & experimental systems

n/a	Involved in the study
☐	☒ Antibodies
☐	☒ Eukaryotic cell lines
☒	☐ Palaeontology and archaeology
☐	☒ Animals and other organisms
☒	☐ Clinical data
☒	☐ Dual use research of concern
☒	☐ Plants

Methods

n/a	Involved in the study
☒	☐ ChIP-seq
☐	☒ Flow cytometry
☒	☐ MRI-based neuroimaging

Antibodies

Antibodies used

All the used antibodies are reported in the manuscript and here. Antibodies dilutions are reported for each antibody in the Methods section or in the Supplementary Table 1 as amount/million of cells for the flow cytometry experiments. Rabbit anti-SMAD3 (#9523, Cell Signaling Technology), rabbit anti-BRD4 (#128874, Abcam), mouse anti-beta actin (#A2228, Sigma-Aldrich), rabbit anti-BRD4 (#A301-985A100, Thermo Fisher Scientific), rabbit anti-IgG isotype control (#2729, Cell Signaling Technology), anti-Rpb1 NTD (RNA Pol II, #149535, Cell Signaling Technology), anti-α-tubulin (#sc8035, Santa Cruz Biotechnologies), rabbit anti-human CD56 (MRQ-42, Cell Marque, Sigma-Aldrich), mouse anti-human CD45 (2B11, PD7/26, Cell Marque, Sigma-Aldrich), anti-human CD45 FITC (#342408, BD), anti-human CD45 PE-Cy™7 (#557748, BD), anti-human CD3 FITC (#300406, Biolegend),

anti-human CD4 APC (#130-113-784, Miltenyi Biotech), anti-human CD8 APC-Vio®770 (#130-110-819, Miltenyi Biotech), anti-human CD14 PE (#342408, BD), anti-human CD19 PE-Vio® 770 (#130-113-170, Miltenyi Biotech), anti-human CD56 FITC (#562794, BD), anti-human CD16 APC-H7 (#560195, BD), anti-human CD94 APC (#559878, BD), anti-human IFN-γ PE (#340452, BD), anti-human CD107a APC (#130-119-869, Miltenyi Biotech), anti-human CD152 PE (#130-118-357, Miltenyi Biotech), anti-human PD-1 APC (#130-117-694, Miltenyi Biotech), anti-human TIGIT PE (#130-116-814, Miltenyi Biotech), anti-human TIM3 APC (#130-120-700, Miltenyi Biotech), anti-human CD223 APC-Vio770 (#130-130-284, Miltenyi Biotech), anti-mouse CD45 PE-Vio770 (#130-117-529, Miltenyi Biotech), anti-mouse CD4 FITC (#553729, BD); anti-mouse CD8 APC-Cy7 (#557654, BD), anti-mouse CD19 APC (#17-0193-80, Thermo Fisher), anti-mouse NK1.1 APC (#17-5941-81, Thermo Fisher), anti-mouse CD335 APC (#130-117-514, Miltenyi Biotech), anti-mouse PD1 APC-Cy7 (#135223, Biolegend), anti-mouse TIGIT PE (#130-120-296, Miltenyi Biotech), anti-mouse CD152 PE (#130-102-570, Miltenyi Biotech), anti-mouse CD107a FITC (#130-102-191, Miltenyi Biotech), anti-mouse CD122 (#BE0298, BioXcell).

Validation

Validation information for primary antibodies can be found at the website of manufacturers, as follows:

Rabbit anti-SMAD3 (#9523, Cell Signaling Technology) (Western blot and ChIP): https://www.cellsignal.com/products/primary-antibodies/smad3-c67h9-rabbit-mab/9523

rabbit anti-BRD4 (#128874, Abcam) (Western blot): https://www.abcam.com/products/primary-antibodies/brd4-antibody-epr51502-ab128874.html

mouse anti-beta actin (#A2228, Sigma-Aldrich) (Western blot): https://www.sigmaaldrich.com/IT/it/product/sigma/a2228

rabbit anti-BRD4 (#A301-985A100, Thermo Fisher Scientific) (ChIP): https://www.thermofisher.com/antibody/product/BRD4-Antibody-Polyclonal/A301-985A100; This antibody was validated for ChIP-seq by the ENCODE project consortium, see: https://www.encodeproject.org/antibodies/ENCAB782ZNQ/

rabbit anti-IgG isotype control (#2729, Cell Signaling Technology) (ChIP): https://www.cellsignal.com/products/primary-antibodies/normal-rabbit-igg/2729

anti-Rpb1 NTD (RNA Pol II, #14958S, Cell Signaling Technology) (Western blot): https://www.cellsignal.com/products/primary-antibodies/rpb1-ntd-d8l4y-rabbit-mab/14958

anti-α-tubulin (#sc8035, Santa Cruz Biotechnologies) (Western blot):
https://www.scbt.com/it/p/alpha-tubulin-antibody-tu-02

rabbit anti-human CD56 (MRQ-42, Cell Marque, Sigma-Aldrich) (IHC): https://www.sigmaaldrich.com/IT/it/product/sigma/156r9

mouse anti-human CD45 (2B11, PD7/26, Cell Marque, Sigma-Aldrich) (IHC): https://www.sigmaaldrich.com/IT/it/product/sigma/145m9

Flow cytometry:

anti-human CD45 FITC (#342408, BD): https://www.bdbiosciences.com/content/dam/bdb/products/global/reagents/flow-cytometry-reagents/clinical-diagnostics/multicolor-cocktails-and-kits-ivd-ce-ivds/342xxx/3424xx/342408_base/pdf/23-5297.pdf

anti-human CD45 PE-Cy™7 (#557748, BD): https://www.bdbiosciences.com/en-it/products/reagents/flow-cytometry-reagents/research-reagents/single-color-antibodies-ruo/pe-cy-7-mouse-anti-human-cd45.557748

anti-human CD3 FITC (#300406, Biolegend): https://www.biolegend.com/nl-be/products/fitc-anti-human-cd3-antibody-863

anti-human CD4 APC (#130-113-784, Miltenyi Biotech): https://www.miltenyibiotec.com/IT-en/products/cd4-antibody-anti-human-reafinity-rea623.html#conjugate=apc:size=100-tests-in-200-ul

anti-human CD8 APC-Vio®770 (#130-110-819, Miltenyi Biotech): https://www.miltenyibiotec.com/IT-en/products/cd8-antibody-anti-human-reafinity-rea734.html#conjugate=apc-vio-770:size=30-tests-in-60-ul

anti-human CD14 PE (#342408, BD): https://www.bdbiosciences.com/content/dam/bdb/products/global/reagents/flow-cytometry-reagents/clinical-diagnostics/multicolor-cocktails-and-kits-ivd-ce-ivds/342xxx/3424xx/342408_base/pdf/23-5297.pdf

anti-human CD19 PE-Vio® 770 (#130-113-170, Miltenyi Biotech): https://www.miltenyibiotec.com/IT-en/products/cd19-antibody-anti-human-lt19.html#conjugate=vioblue:size=100-tests-in-200-ul
anti-human CD56 FITC (#562794, BD): https://www.bdbiosciences.com/en-it/products/reagents/flow-cytometry-reagents/research-reagents/single-color-antibodies-ruo/fitc-mouse-anti-human-cd56-ncam-1.562794
anti-human CD16 APC-H7 (#560195, BD): https://www.bdbiosciences.com/en-it/products/reagents/flow-cytometry-reagents/research-reagents/single-color-antibodies-ruo/apc-h7-mouse-anti-human-cd16.560195
anti-human CD94 APC (#559876, BD): https://www.bdbiosciences.com/en-it/products/reagents/flow-cytometry-reagents/research-reagents/single-color-antibodies-ruo/apc-mouse-anti-human-cd94.559876
anti-human IFN-γ PE (#340452, BD): https://www.bdbiosciences.com/en-it/products/reagents/flow-cytometry-reagents/clinical-discovery-research/single-color-antibodies-ruo-gmp/pe-mouse-anti-human-ifn.340452
anti-human CD107a APC (#130-119-869, Miltenyi Biotech): https://www.miltenyibiotec.com/IT-en/products/cd107a-lamp-1-antibody-anti-human-h4a3.html#conjugate=vioblue:size=100-tests-in-200-ul
anti-human CD152 PE (#130-118-357, Miltenyi Biotech): https://www.miltenyibiotec.com/IT-en/products/cd152-antibody-anti-human-bni3.html#conjugate=pe:size=100-tests-in-200-ul
anti-human PD-1 APC (#130-117-694, Miltenyi Biotech): https://www.miltenyibiotec.com/IT-en/products/cd279-pd1-antibody-anti-human-pd1-3-1-3.html#conjugate=apc:size=100-tests-in-200-ul
anti-human TIGIT PE (#130-116-814, Miltenyi Biotech): https://www.miltenyibiotec.com/IT-en/products/tigit-antibody-anti-human-reafinity-rea1004.html#conjugate=pe:size=100-tests-in-200-ul
anti-human TIM3 APC (#130-120-700, Miltenyi Biotech): https://www.miltenyibiotec.com/IT-en/products/tim-3-antibody-anti-human-f38-2e2.html#conjugate=apc:size=100-tests-in-200-ul
anti-human CD223 APC-Vio770 (#130-130-284, Miltenyi Biotech): https://www.miltenyibiotec.com/IT-en/products/cd223-antibody-anti-human-reafinity-rea351.html#conjugate=apc-vio-770:size=100-tests-in-200-ul
anti-mouse CD45 PE-Vio770 (#130-117-529, Miltenyi Biotech): https://www.miltenyibiotec.com/IT-en/products/cd45-antibody-anti-mouse-30f11.html#conjugate=pe-vio-770:size=30-ug-in-200-ul
anti-mouse CD4 FITC (#553729, BD): https://www.bdbiosciences.com/en-it/products/reagents/flow-cytometry-reagents/research-reagents/single-color-antibodies-ruo/fitc-rat-anti-mouse-cd4.553729
anti-mouse CD8 APC-Cy7 (#557654, BD): https://www.bdbiosciences.com/en-it/products/reagents/flow-cytometry-reagents/research-reagents/single-color-antibodies-ruo/apc-cy-7-rat-anti-mouse-cd8a.557654
anti-mouse CD19 APC (#17-0193-80, Thermo Fisher): https://www.thermofisher.com/antibody/product/CD19-Antibody-clone-eBio1D3-1D3-Monoclonal/17-0193-82
anti-mouse NK1.1 APC (#17-5941-81, Thermo Fisher): https://www.thermofisher.com/antibody/product/NK1-1-Antibody-clone-PK136-Monoclonal/17-5941-81
anti-mouse CD335 APC (#130-117-514, Miltenyi Biotech): https://www.miltenyibiotec.com/IT-en/products/cd335-nkp46-antibody-anti-mouse-29a1-4-9.html#conjugate=apc:size=30-ug-in-200-ul
anti-mouse PD1 APC-Cy7 (#135223, Biolegend): https://www.biolegend.com/en-gb/search-results/apc-cyanine7-anti-mouse-cd279-pd-1-antibody-9742?GroupID=BLG7927
anti-mouse TIGIT PE (#130-120-296, Miltenyi Biotech): https://www.miltenyibiotec.com/IT-en/products/tigit-antibody-anti-mouse-reafinity-rea536.html#conjugate=pe:size=30-ug-in-200-ul
anti-mouse CD152 PE (#130-102-570, Miltenyi Biotech): https://www.miltenyibiotec.com/IT-en/products/cd152-antibody-anti-mouse-uc10-4b9.html#conjugate=pe:size=30-ug-in-1-ml
anti-mouse CD107a FITC (#130-102-191, Miltenyi Biotech): https://www.miltenyibiotec.com/IT-en/products/cd107a-lamp-1-antibody-anti-mouse-1d4b.html#conjugate=fitc:size=30-ug-in-1-ml
anti-mouse CD122 (#BE0298, BioXcell)(in vivo mice treatments): https://bioxcell.com/invivomab-anti-mouse-cd122-il-2rb-be0298

Eukaryotic cell lines

Policy information about cell lines and Sex and Gender in Research

Cell line source(s)	NCI-H23 (Human, male), NCI-H1299 (Human, male), and NCI-H1975 (Human, female) cell lines were obtained from Dr. Massimo Broggini (IRCCS-Istituto di Ricerche Farmacologiche Mario Negri, Milan, Italy). LLC1 (Mouse) was obtained from Dr. Francesco Bertolini (IFOM-IEO Campus, Milan, Italy). HEK293T (ATCC CRL-3216, Human, female) and NK92® (ATCC CRL-2407, Human, male) cell line was purchased from ATCC (LGC Standards).
Authentication	All cell lines have been authenticated through SNP or STR profiling by Multiplexion Gmbh (Heidelberg, Germany).
Mycoplasma contamination	All cell lines are routinely checked (i.e. every 2 weeks) for mycoplasma contamination through MycoAlert Mycoplasma Detection kit (#LT07-318, Lonza). In case Mycoplasma was detected, the batch was discarded and a new one was thawed from liquid nitrogen stock.
Commonly misidentified lines (See ICLAC register)	We check the ICLAC register of communly misidentified cell lines and none of the cell lines we used in this manuscript are present in the register.

Animals and other research organisms

Policy information about studies involving animals; ARRIVE guidelines recommended for reporting animal research, and Sex and Gender in Research

Laboratory animals	Nonobese diabetic severe combined immunodeficient (NOD/SCID) interleukin-2 receptor γ (IL-2Rg)-null (NSG) mice and C57BL/6 mice. Age of NOD/SCID mice was 6-8 weeks, age of C57BL/6 mice was 6 weeks.

Wild animals	The study did not involve wild animals.
Reporting on sex	The study design was not restrained to a specific sex. The study applied both male and female mice according to distinct management purposes, but it did not involve sex-related findings. Mice were detected and managed by highly specialized technicians and two Veterinarians available in animal facilities at the European Institute of Oncology–Italian Foundation for Cancer Research (FIRC) Institute of Molecular Oncology (IEO–IFOM, Milan, Italy).
Field-collected samples	No field-collected samples were used in this study.
Ethics oversight	Italian Ministry of Health (Authorization code 780/2020-PR).

Note that full information on the approval of the study protocol must also be provided in the manuscript.

Plants

Seed stocks	*Report on the source of all seed stocks or other plant material used. If applicable, state the seed stock centre and catalogue number. If plant specimens were collected from the field, describe the collection location, date and sampling procedures.*
Novel plant genotypes	*Describe the methods by which all novel plant genotypes were produced. This includes those generated by transgenic approaches, gene editing, chemical/radiation-based mutagenesis and hybridization. For transgenic lines, describe the transformation method, the number of independent lines analyzed and the generation upon which experiments were performed. For gene-edited lines, describe the editor used, the endogenous sequence targeted for editing, the targeting guide RNA sequence (if applicable) and how the editor was applied.*
Authentication	*Describe any authentication procedures for each seed stock used or novel genotype generated. Describe any experiments used to assess the effect of a mutation and, where applicable, how potential secondary effects (e.g. second site T-DNA insertions, mosiacism, off-target gene editing) were examined.*

Flow Cytometry

Plots

Confirm that:

- [x] The axis labels state the marker and fluorochrome used (e.g. CD4-FITC).
- [x] The axis scales are clearly visible. Include numbers along axes only for bottom left plot of group (a 'group' is an analysis of identical markers).
- [ ] All plots are contour plots with outliers or pseudocolor plots.
- [x] A numerical value for number of cells or percentage (with statistics) is provided.

Methodology

Sample preparation	Tissues were enzymatically digested with Liberase™ DH Research grade (Sigma-Aldrich, 0.26 IU/ml) for 1 hour at 37°C. The cell suspension was filtered through a 40µm strainer to remove tissue debris. Red Blood Cell Lysis Buffer (Sigma-Aldrich) was used to remove erythrocytes. Cells were resuspended in cold FACs Buffer (1x PBS, 5mM EDTA, 2% BSA) and stained with the antibodies listed in Supplementary Table 1.
Instrument	BD FACS CANTO II (BD)
Software	BD FACSDIVA Software (BD), Kaluza v 2.1 Software (Beckman Coulter).
Cell population abundance	Approximately 100.000 events were acquired for each sample. No flow cytometry-based cell sorting was performed in this study.
Gating strategy	Forward versus side scatter (FSC vs SSC) gating was used to identify cells based on size and granularity. After the exclusion of doublets (FSC-A vs FSC-H) and dead cells (7AAD+), immune cells were selected for CD45+ expression. The main markers were used to gate T-lymphocytes (CD3+), B-lymphocytes (CD19+), NK cells (CD56+), and Monocytes (CD14+). T-lymphocytes (CD3 +) were subsequently classified as T-helper (CD4+) or cytotoxic (CD8+) T cells. Negative controls with unstained cells were acquired and analyzed within each experiment or sample to properly set gating axes and identify negative cells.

- [x] Tick this box to confirm that a figure exemplifying the gating strategy is provided in the Supplementary Information.
